# Cytotoxic and antimicrobial activities of two new sesquiterpenoids from red sea brittle star *Ophiocoma dentata*

**DOI:** 10.1038/s41598-022-12192-7

**Published:** 2022-05-17

**Authors:** Shaymaa E. El Feky, Mohamed S. M. Abd El Hafez, Nadia A. Abd El Moneim, Hassan A. H. Ibrahim, Mohamed A. Okbah, Athar Ata, Amel S. El Sedfy, Ahmed Hussein

**Affiliations:** 1 Radiation Sciences Department, Medical Research Institute, University of Alexandria, Alexandria, Egypt; 2 National Institute of Oceanography and Fisheries, NIOF, Cairo, Egypt; 3 Cancer Management and Research Department, Medical Research Institute, Alexandria University, Alexandria, Egypt; 4 Department of Chemistry, University of Winnipeg, Winnipeg, MB Canada; 5 Pathology Department, Medical Research Institute, University of Alexandria, Alexandria, Egypt; 6 Department of Biotechnology, Institute of Graduate Studies and Research, Alexandria University, Bab Sharqi, Egypt

**Keywords:** Biotechnology, Cancer, Ocean sciences

## Abstract

Bioactive compounds were extracted from a locally available brittle star; *Ophiocoma dentata*, collected from the Red Sea, Egypt. Two new sesquiterpenoids; 8, 11-epoxy-9(15)-himachaladiene-4-ol (O8-ophiocomane) and, 11-epoxy-9(15)-himachaladiene-4-ol (O7-ophiocomane) were isolated and characterized using appropriate techniques. Structure elucidation was estimated via 1D NMR, 2D NMR, FT-IR and mass spectroscopy analyses. The isolated compounds were tested for cytotoxic, antibacterial and antifungal activities. Pure compounds showed a dose dependent reduction in MCF-7 cells viability with LC50 of 103.5 and 59.5 μg/ml for compounds 1 and 2 respectively compared to the chemotherapeutic drug cisplatin (47.4 µg/ml). In vivo experiments showed that *O. dentate* extract significantly reduced tumor progression and improved hematological parameters and liver functions of tumor-bearing mice when administered either before or after tumor cells’ injection. The most remarkable antimicrobial effects of *O. dentate* crude extract were against *Staphylococcus aureus*, *Vibrio damsela* and *Pseudomonas* *aeruginosa* while the pure compounds showed activity against *P. aeruginosa* alone. Neither the crude extract nor the pure compounds have shown activity against *Aeromonas hydrophila*. These results indicates that *O. dentata* extract and newly isolated compounds have shown a promising cytotoxic, antiproliferative and antimicrobial activities that might be useful for pharmaceutical applications.

## Introduction

Since the 1940’s, marine organisms have drawn much attention as a source of bioactive compounds with potential therapeutic applications^1^. The diversity of the marine environment resulted in the discovery of structurally unique compounds. Enormous research in the past decades lead to the discovery many of compounds with antimicrobial, antioxidant and anticancer properties from marine origin^2^. This is due to the lack of physical defense mechanism, which equipped these organisms with natural toxic compounds to survive predation^3^. Nevertheless, many marine organisms are yet to be explored.

Ophiuroidea (brittle stars) is the largest class of echinoderms having a starfish-resembling body outline with 5 locomotive arms originating from a relatively small central disk^4^. There are accumulating evidence echinoderms are a source of secondary metabolites with potential useful therapeutic properties. However, the research on Ophiurodea-derived metabolites is relatively limited in comparison to other echinoderms^5^. Polar steroids make up the bulk of secondary metabolites found in brittle stars. Also isolated were naphthaquinones, terpenes, phenylpropanoids, carotenoids and cerebrosides^6^. The diversity of theses metabolites encourages more search for their pharmacological properties and potential applications^7^.

Cancer has been the world's biggest cause of mortality over the years and the main cause of decreasing life expectancy^8^. Cancer is a complicated illness caused by the interaction of genetic and environmental variables, and it is defined by uncontrolled growth and spread of cells that have the capacity to avoid cell death, despite their molecular defects boosting tumor invasiveness, proliferation and angiogenesis^9^. Furthermore, different forms of cancer develop resistance to common therapeutic options including chemo and radiotherapy. These are compound with the toxicities and side effects associated with increasing doses of these treatments. This necessitates the search for innovative anti-tumor chemicals capable of overcoming these issues^10^. The search continues for promising new agents that show cytotoxic activities that can boost the efficacy of traditional treatments. Therefore natural products, including those extracted from marine organisms appear to be an appealing source for the creation of novel medications in this area, owing to their capacity to reach several targets with little side effects and their potency against a variety of cancer types^11^. In addition to their promising cytotoxic and anticancer properties, many marine originated compounds have also shown a promising role as antimicrobial, antifungal and anti-inflammatory agents with beneficial health applications^12^.

Despite the extensive research on marine organisms worldwide, the work on the unique ecosystem of the Red sea organisms is still in its preliminary stages. A large number of marine organisms have been identified^13^, however, there’s still limited data on their biological composition as well as bioactivities and potential applications especially in the pharmaceutical and medical field. To the best of our knowledge, no previous investigations of the potential bioactivities of *O. dentata* habituated in the Egyptian Red Sea have been published to date. Therefore, the present study was suggested to reveal the isolation of bioactive compounds from brittle star; *O. dentata* and explore their potential bioactive properties and future medical applications.

## Results

### Characterization of collected water samples

The hydro-chemical and physical properties of collected sea water samples from the study area are illustrated in Table 1.

**Table 1:** The hydro-chemical and physical properties of collected sea water samples.

Parameter	Measurements
Temperature	28.3 °C
pH	8.4
Dissolved oxygen (DO)	5.8 ml O_2_/l
Oxidizable organic matter (OOM)	2.66 mg
Salinity	41.6%
Calcium	512 mg/l
Alkalinity	3.4 meq/l

### Biochemical analysis of *O. dentata* samples

The biochemical composition of *O. dentata* samples including humidity, ash, organic matter, nitrogen, proteins, lipids, fibers and carbohydrates percentages were analyzed and the summarized in Table 2.

**Table 2:** Biochemical composition of *O. dentata* samples.

Parameter	Measurements
Humidity (%)	18
Ash (%)	11
Organic matter (%)	71
Nitrogen (%)	1.6
Protein (%)	9.8
Lipids (%)	3.7
Fibers (%)	4.9
Carbohydrates (%)	47.4

### Extraction and isolation of bioactive compounds from brittle star

**Figure 1:**
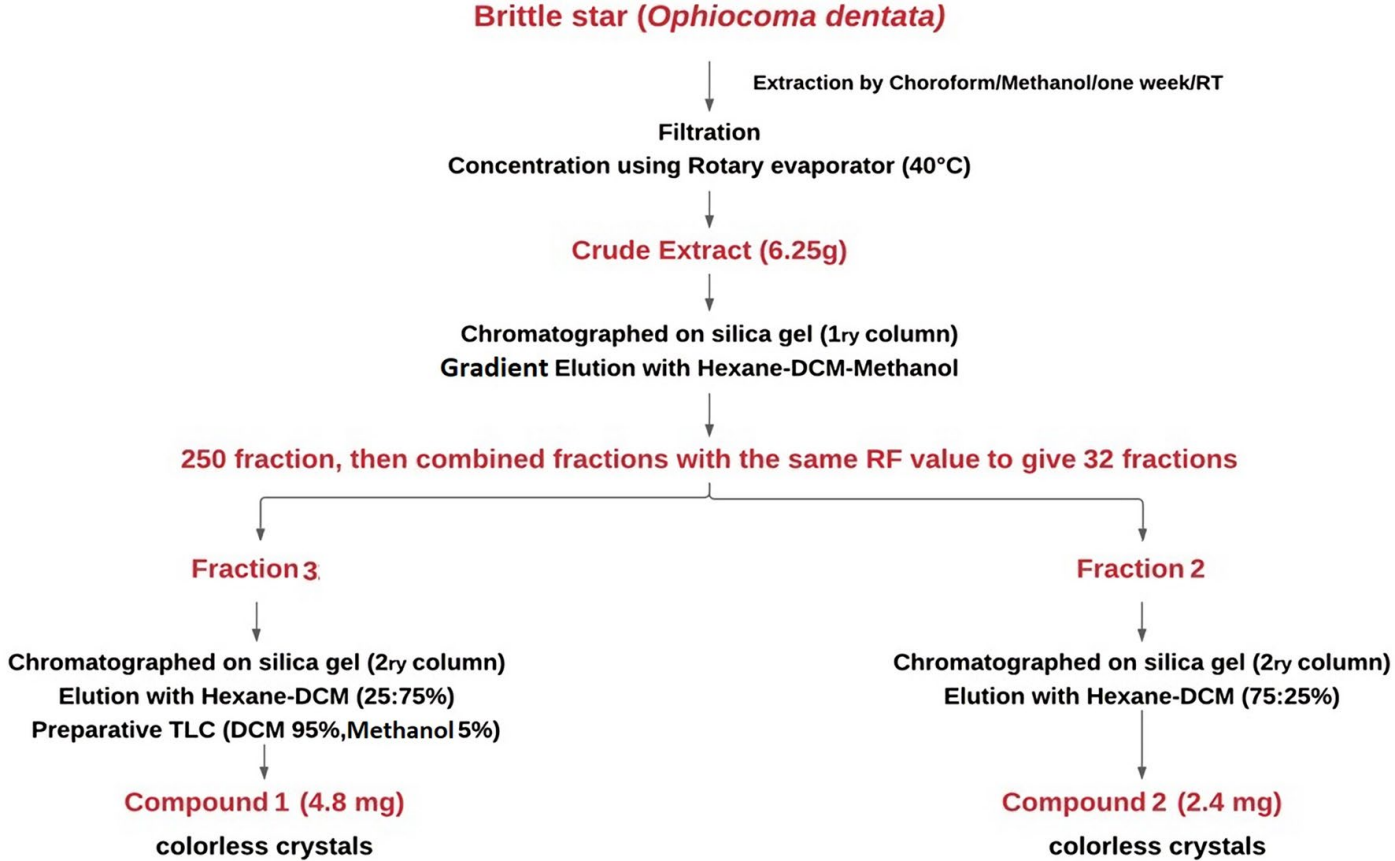
Flow chart pure compounds’ extraction from *O. dentate.*

**Figure 2:**
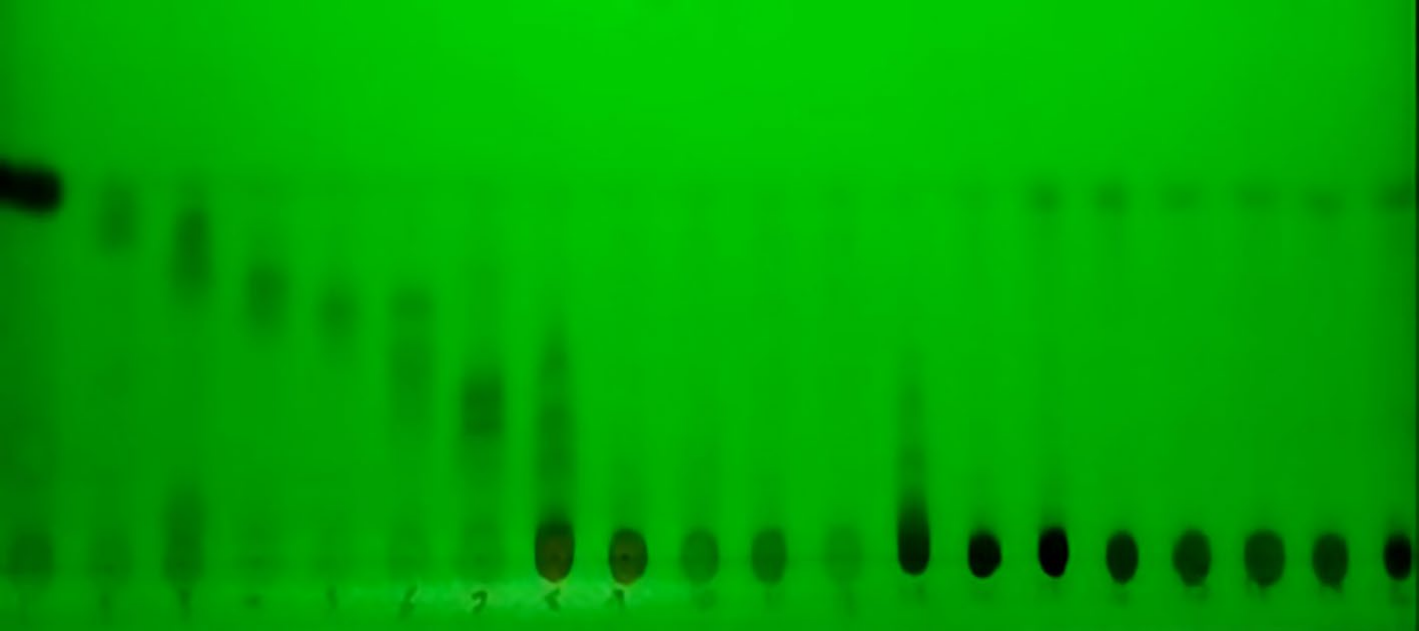
The 250 fractions combined (with the same RF value) fractions.

The extraction flow chart and isolation of the pure compounds from *O. dentata* are shown in Fig. 1. A 14.62 g sample of *O. dentata* was extracted using chloroform and methanol individually with 1:2 weight to volume ratio then the resultant extracts were combined with 1:1 weight to weight ratio. The crude extract was then subjected to primary chromatography column with 60–200 µm silica gel (70–230 mesh) using a gradient elution technique using Hexane-Dichloromethane (DCM)-Methanol and the eluted sample was collected as 250 fractions. Thin layer chromatography (TLC) was then performed for all fractions and each TLC plate was examined under UV detector lamp, the spots were marked, then the plate was sprayed with 10% sulfuric acid and dried with hot air or by hot plate. Fractions with the same RF value were combined to give 32 fractions out of 250 (Fig. 2). All fractions were dried and collected in clean vials and two of which were further subjected to further analysis. One of the fractions was chromatographed on silica gel (2ry column) using Hexane:DCM (25:75%) as a mobile phase in ascending chromatography followed by descending chromatography on TLC using DCM: Methanol (95:5 %) as a mobile phase to obtain colorless crystals referred to as Compound 1. The second fraction was chromatographed on silica gel using Hexane:DCM (75:25%) as a mobile phase to obtain the colorless crystals referred to as Compound 2.

### Structure elucidation of the isolated pure compounds

#### Characterization of Compound 1 (O8-ophiocomane)

**Figure 3:**
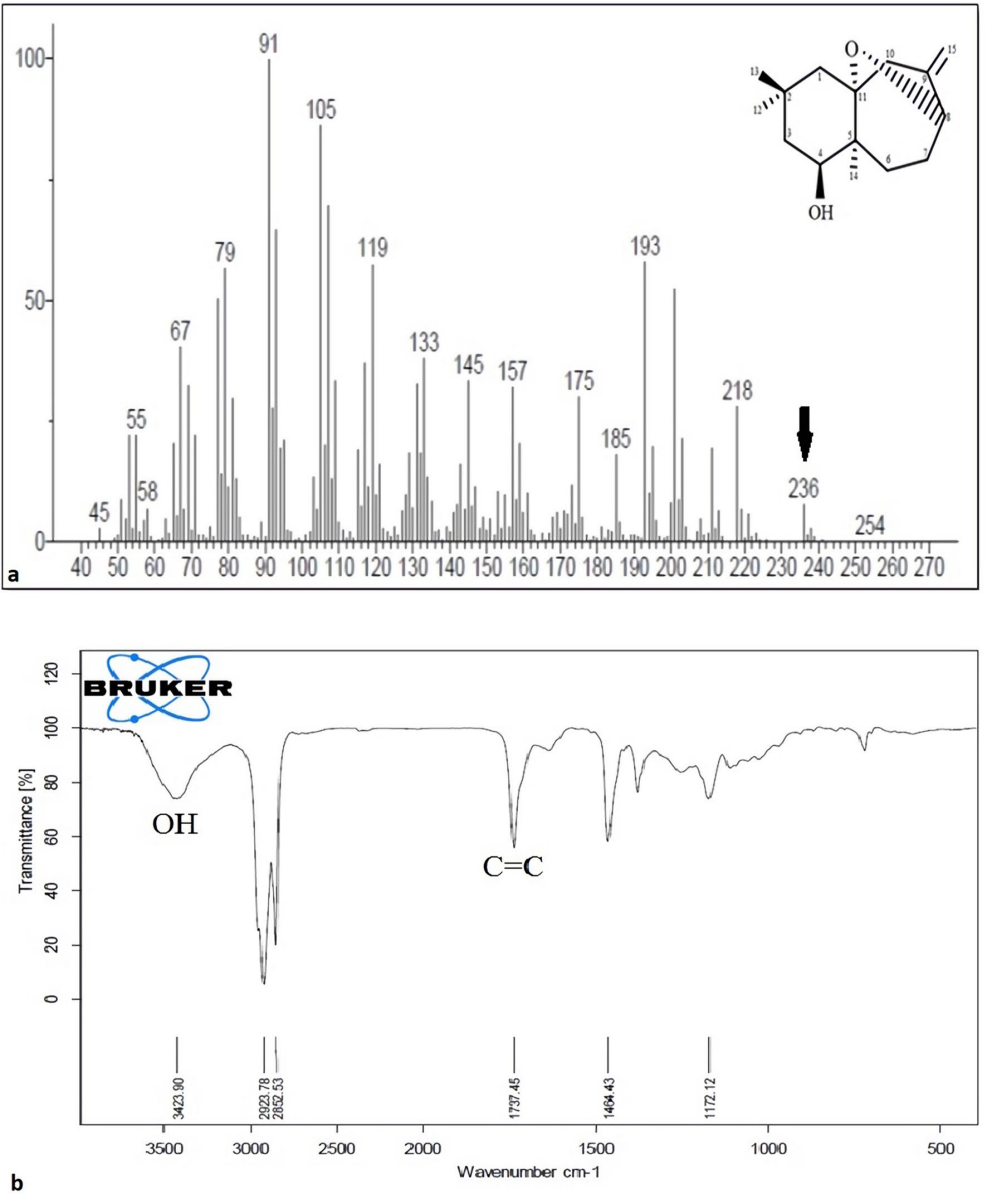
The (**a**) GC/MS and (**b**) FT-IR spectra of compound 1 (O8-Ophiocomane).

Compound 1 is a novel colorless crystallized compound. GC/MS (m/z 236.3420) determines its chemical formula as C15H24O2, necessitating four degrees of unsaturation. The hydroxyl (3423 cm^−1^) and C=C double bond (1737 cm^−1^) groups were seen in the IR spectra (Fig. 3). Three single methyl [H 0.98 (3H, s, H-12), 1.28 (3H, s, H-13), 1.19 (3H, s, H-14)] and two terminal olefinic protons [H 5.19 and H 4.85 (each 1H, s, H-15a and H-15b)] were found in the 1H NMR spectroscopic data. Three methyl, six methylene (including one terminal C=C double bond), two oxygenated methine, and four quaternary carbons were identified in the 13C NMR spectroscopic data (including one oxygenated sp3 quaternary carbon, one sp2 quaternary carbon). The 1H and 13C NMR spectra of component 1 revealed the presence of sesquiterpenoids.

A comprehensive study of 1D and 2D NMR spectra, including HSQC, HMBC, and COSY spectra, was used to assign the whole NMR spectroscopic data of compound 1. The essential spin systems were revealed by the COSY correlations of H-3/H-4 and H-6/H-7/H-8. The correlations from H-12 and H-13 to C-1, C-2, and C-3 in the HMBC spectrum indicated that the 12-CH3 and 13-CH3 were both connected to C-2; the correlations from H-14 to C-4, C-5, C-6, and C-11 suggested that the 14-CH3 was linked to C-5; and the correlations from H-15a and H-15b to C-8, C-9, and C-10 confirmed the presence of the molecule's terminal double bond, which was determined The downfield chemical shifts of the two oxymethine carbons and one oxygenated quaternary carbon, as well as the HMBC and COSY correlations, were used to assign the two oxymethine carbons and one oxygenated quaternary carbon to C-4, C-8, and C-11, respectively. The remaining unsaturation in 5 was supposed to be an oxygen bridge between C-11 and C-4 or C-11 and C-8, establishing another ring system, aside from one double bond and two carbon ring systems. The oxygen bridge was found to be between C-11 and C-8, producing a cage-like structure, based on the HMBC correlation from H-8 to C-11.

NOESY tests were used to determine the relative configuration of compound 1. The correlations of H-14/H-4 and H-14/H-12 in the NOESY spectrum suggested that 14-CH3, 12-CH3, and H-4 were on the same side of the ring and had R-orientation. H-8 and H-10a (H 3.92) had a significant connection, indicating that the oxygen bridge was on the same side. Based on the correlations of H-10a/H-13 and H-10a/H-8 as depicted in the molecular model, H-8 was found to be -oriented. Figure 3 depicts both 1D and 2D NMR spectra, as well as FT-IR and GC/MS data (a–h). As a result, the structure of compound 1 was determined, and the compound was given the designation 8, 11-epoxy-9(15)-himachaladiene-4-ol (O8-ophiocomane).

#### Characterization of compound 2 (O7-ophiocomane)

**Figure 4:**
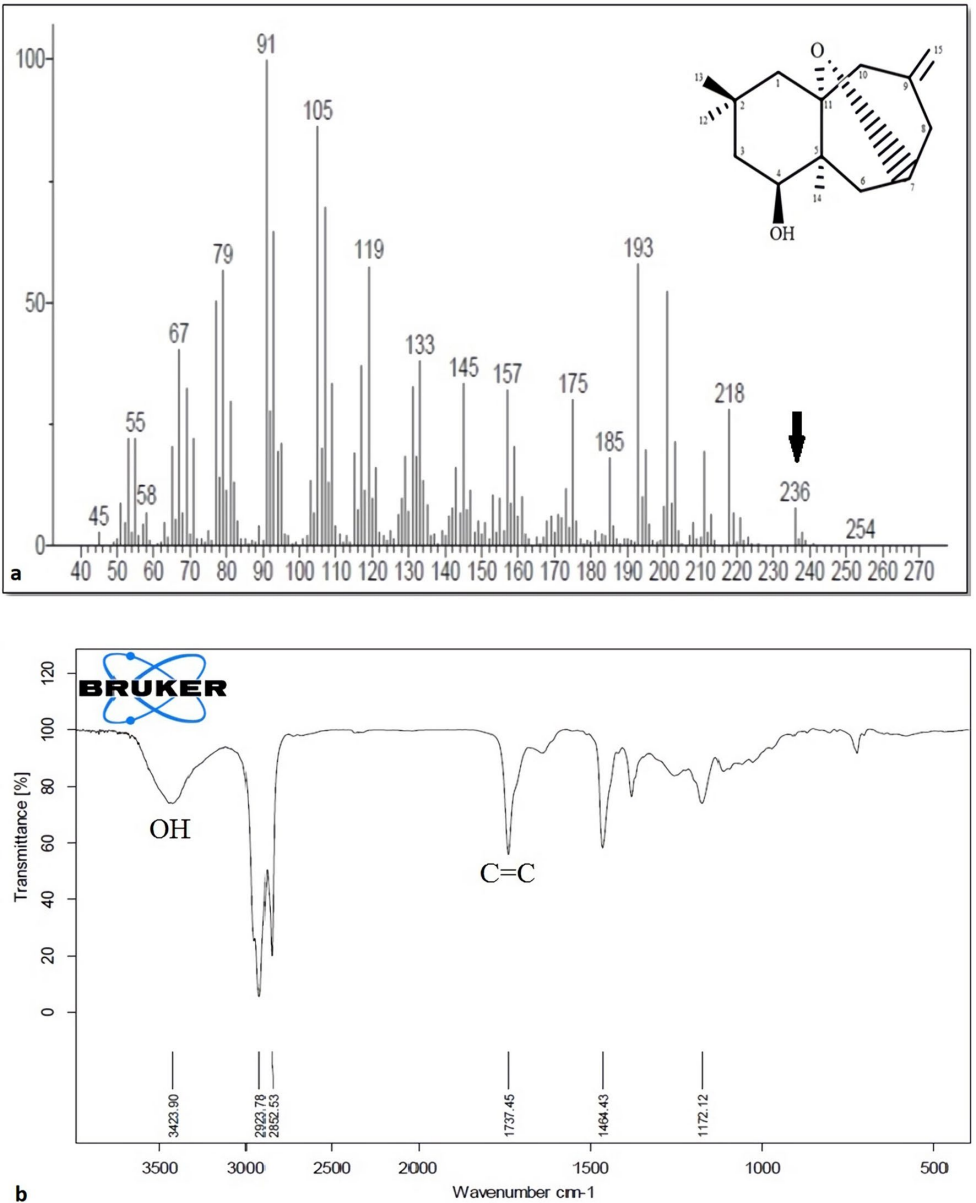
The (**a**) GC/MS and (**b**) FT-IR spectra of compound 2 (O7-Ophiocomane).

Compound 2 is a novel colorless crystallized compound. GC/MS (m/z 236.3420) determined its chemical formula as C15H24O2, necessitating four degrees of unsaturation. The hydroxyl (3423 cm^−1^) and C=C double bond (1737 cm^−1^) groups were seen in the IR spectra (Fig. 4). The only difference between compound 2 and compound 1 is the oxygen bridge between C-11 and C-7. Along with IR and Mass spectra, NMR spectra verified this. Figure 4 shows 1H and 13C NMR data (a–h). As a result, the structure of compound 2 was determined, and the compound was given the designation 7, 11-epoxy-9(15)-himachaladiene-4-ol (O7-ophiocomane).

Both of the isolated compounds are uncommon in a marine organism and first encountered in brittle star species. The characteristic Sesquiterpenoids isolated from *O. dentata* for first time to be reported according to science finder data base (https://scifinder.cas.org/scifinder/view/scifinder/scifinderExplore.jsf).

### Cytotoxicity and anticancer activity

#### Ophiocoma dentata cytotoxicity against MCF-7 cell line

**Figure 5:**
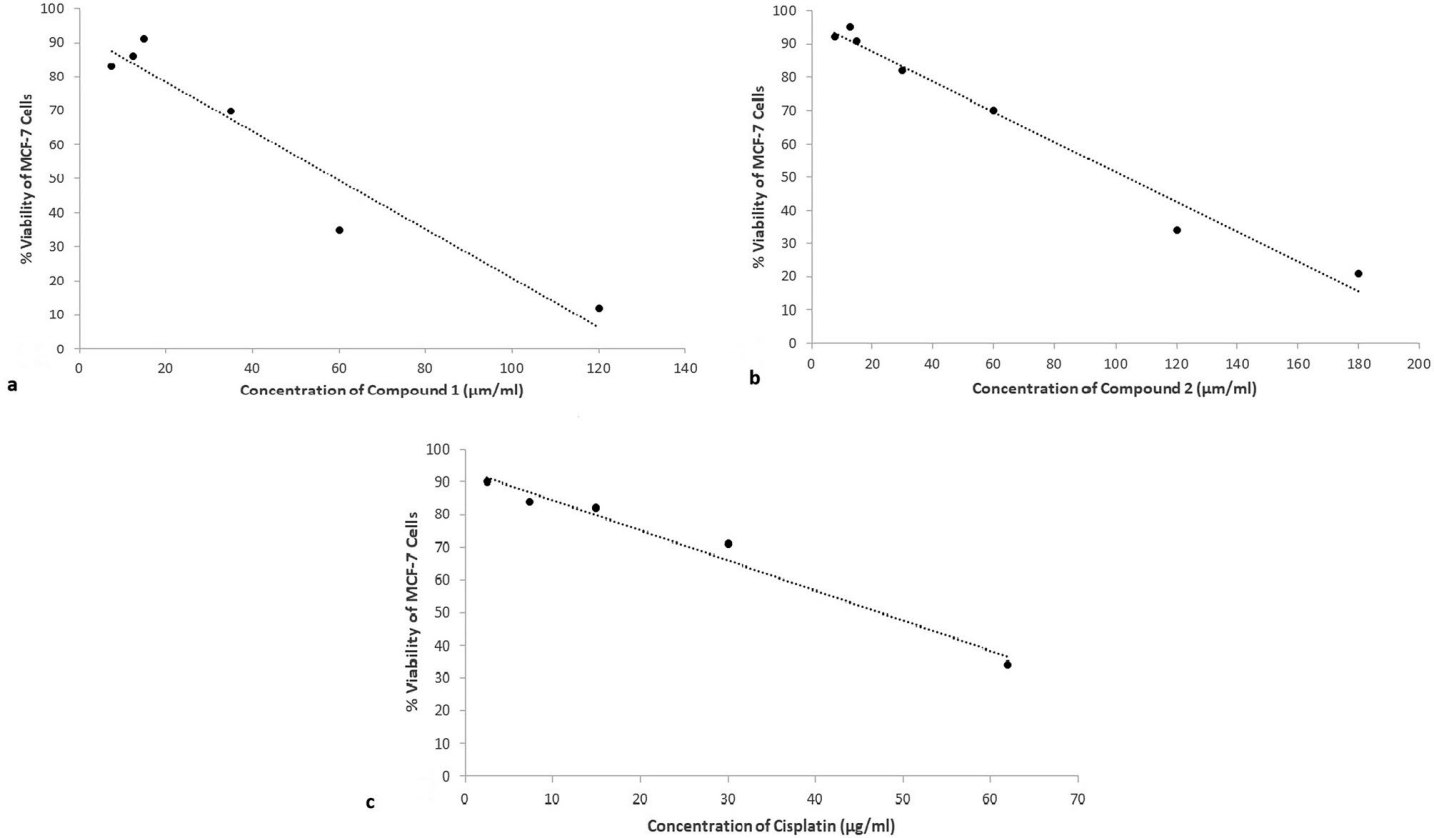
Cytotoxicity of *O. dentata* against MCF-7 cells: the graphs shows the cytotoxicity of (**a**) compound 1 and (**b**) compound 2 purified from *O. dentata* compared to (**c**) cisplatin.

The MTT test was used to assess the cytotoxic effects of *O. dentata* purified compounds 1 and 2 as well as chemotherapeutic drug cisplatin. MCF-7 cells were divided into control and treatment. MTT findings showed that *O. dentata* purified compounds and cisplatin suppressed cell growth in a dose dependent manner. The LC_50_ of pure compounds **1** and **2** were 103.5 and 59.5 μg/ml, respectively, whereas Cisplatin had LC_50_ as 47.4 µg/ml (Fig. 5).

#### Ophiocoma dentata cytotoxic effects against ehrlich tumor-bearing mice

**Figure 6:**
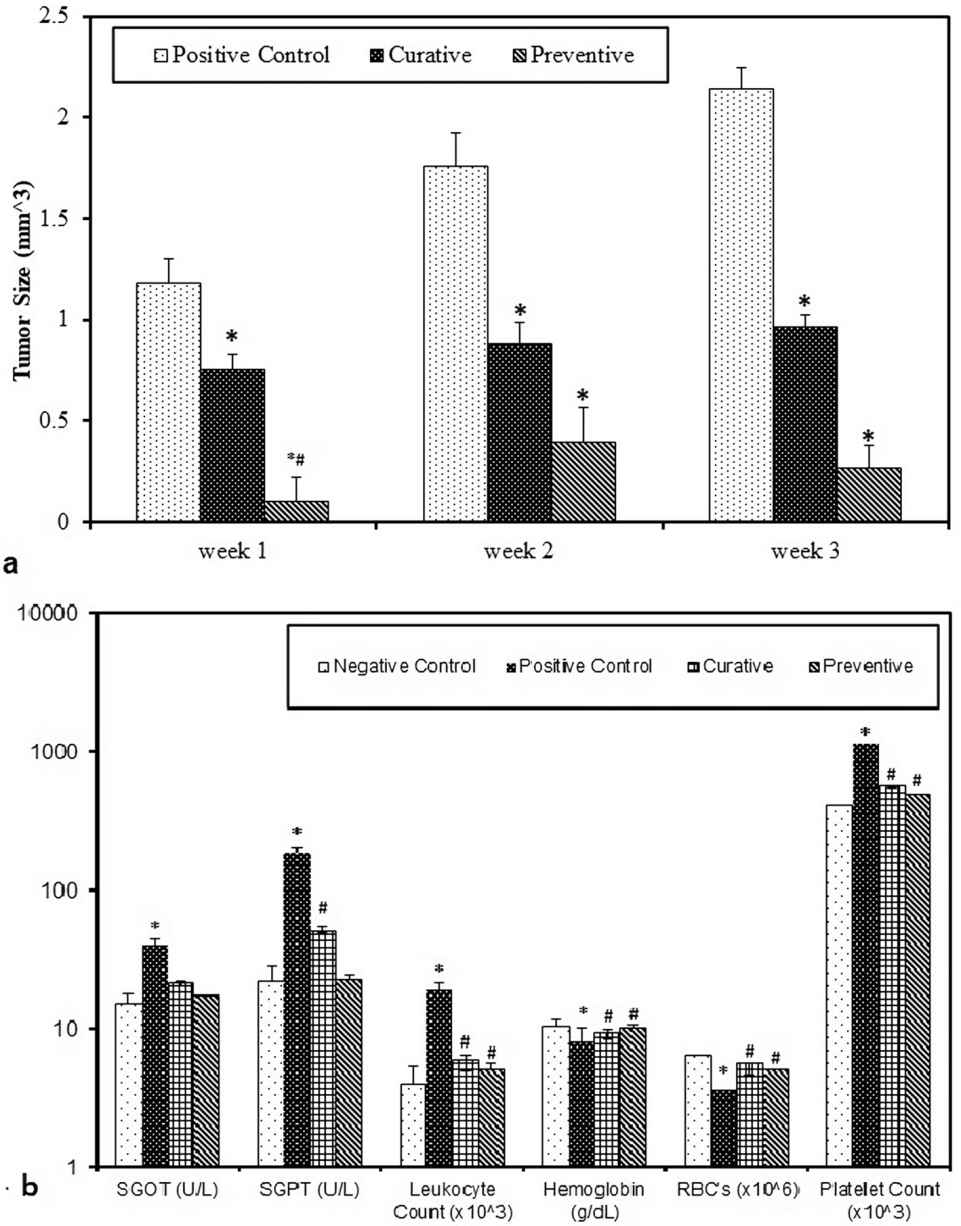
In vivo biological effects of *O. dentata* in Ehrlich tumor-bearing mice including (**a**) tumor size measurements (**b**) hematological parameters and liver function tests in study groups.

Ehrlich tumor-bearing mice were divided into 4 groups, designated as follows: negative control, positive control, preventive and curative groups. Tumor size was measured weekly by caliper for up to 3 weeks till autopsy. Both preventive and curative groups exhibited a significant reduction in tumor mass size when compared to positive control group up to 3 weeks post tumor injection (*p *< 0.001 for preventive group, *p *= 0.05 for curative group at one, two and three weeks). Although preventive group showed a lower mean tumor size compared to curative group, the difference was only significant at first week (*p *= 0.019, 0.061, 0.054 for weeks 1 to 3 respectively) **(**Fig. 6a).

**Figure 7:**
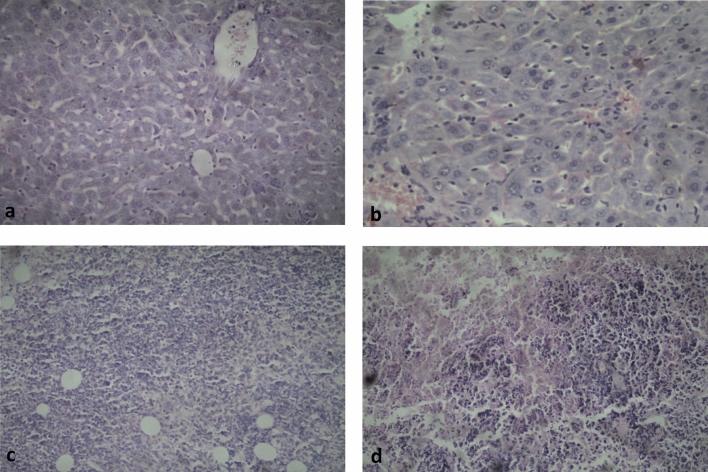
Histopathological analysis of liver in *vivo* study using laboratory animal for cytotoxicity and anticancer activity, where picture (**a**) liver of a healthy control mouse, (**b**) liver of tumor-bearing mouse treated with O. *dentata* extract with no abnormal changes. (**c)** Ehrlich tumors of untreated mouse. (**d**) Ehrlich tumor of *O. dentata* treated mouse showing deeply chromatic pleomorphic nuclei with wide areas of necrosis.

Ehrlich tumor-bearing mice showed a significant elevation in liver functions, leukocytes and platelets counts and a significant reduction in RBC’s and hemoglobin level compared to negative control group (*p *< 0.001). Treatment with crude extract before tumor injection (preventive group) lead to a significant reduction in liver function parameters (*p *< 0.001), leukocyte and platelets counts (*p *< 0.001) and a significant elevation in RBC’s (*p *= 0.04) and hemoglobin levels (*p *< 0.001) compared to positive control group. The curative group showed a significant difference from the positive control groups regarding SGOT (*p *= 0.049), leukocyte (*p *= 0.032), platelets (*p *= 0.030) and red blood cells counts (*p *= 0.001) (Figure 6b). Histopathological analysis of Ehrlich tumors showed deeply chromatic pleomorphic nuclei with wide areas of necrosis in mice treated with *O. dentata* crude extract compared with untreated tumor-bearing mice, however, liver showed no abnormal changes comparted to the normal structure of liver in negative control group (Fig. 7).

#### Ophiocoma dentata antimicrobial activity

The zones of inhibition in different bacterial strains against crude extract and pure compounds were measured and antibacterial activities were expressed as absolute activity unit (AU). The maximum antibacterial activities for *O. dentata* crude extract was recorded against *Staphylococcus aureus* (7.1 ± 0.03 AU) followed by (5.4 ± 0.01 AU) against *Vibrio damsela*, (3.7 ± 0.07 AU) against *Pseudomonas aeruginosa,* (1.8 ± 0.07 AU) against Escherichia *coli,* and (1.4 ± 0.02 AU) against *Enterococcus faecalis.* On the other side, the pure compounds **1** and **2** showed activity (2.8 ± 0.05 and 2.25 ± 0.04 AU), respectively against *P. aeruginosa* alone. Neither the crude extract nor the pure compounds have shown activity against *Aeromonas hydrophila*. Additionally, no inhibition zones have been recorded for the antifungal activity of neither the crude extract nor the pure compounds against *Candida albicans*. The AU values of all compounds are presented in Table 3.

**Table 3:** Screening the anti-bacterial and anti-fungal activity for the *O. dentate* crude extract and pure compounds **1** and **2**.

	Activity unit (AU)		
	Crude extract	Compound 1	Compound 2
Indicator bacterium			
*S. aureus*	7.1 ± 0.03	ND	ND
*V. damsela*	5.4 ± 0.01	ND	ND
*P. aeruginosa*	3.7 ± 0.07	2.8 ± 0.05	2.25 ± 0.04
*E. coli*	1.8 ± 0.07	ND	ND
*E. faecalis*	1.4 ± 0.02	1.8 ± 0.01	1.36 ± 0.04
*A. hydrophila*	ND	ND	ND
Indicator fungi			
*C. albicans*	ND	ND	ND

*ND* No data observed.

## Discussion

Extensive research has been conducted in order to find new safe pharmaceuticals that will have the least negative influence on the body. Researchers in this field have been successful in discovering some alternative-medicine generated from natural resources. Because of their anti-inflammatory, antimicrobial, and antiproliferative properties, medicines of marine origin have long been regarded as the most promising prospects for treating acute and chronic disorders. Numerous compounds been tested and proven to have the potential to operate as an active medication^14^.

Brittle stars (Ophiuroids) come in a variety of shapes and sizes and may be found in a variety of marine habitats^15^. Many drug discovery research have focused on various types of brittle stars found all over the world in order to find compounds with anti-bacterial, anti-fungal, anti-inflammatory, anti-diabetic, and anti-tumor properties^16,17^. *O. dentata* samples were collected from Hurgada coast of the Red Sea. Water from the same area were collected and tested for its hydro-chemical and physical properties as they greatly affect the bio composition of marine organisms^18^. As it was expected, higher temperatures were observed during summer time which can result in extensive water evaporation and low water flow, which by time may lead to accumulation of organic matter and depletion of dissolved oxygen. Higher temperature resulted in up-regulation of metabolism, enhanced movement speed, and increased regeneration rates in the brittle star *Ophiura* *ophiura*, as well as up-regulation of metabolism in the Arctic brittle star *Ophiocten* *sericeum*, according to Wood et al.^19^. The pH of sea water was on the alkaline side 8.4, which may be attributed to the photosynthetic activities of aquatic planktons during daylight. Also, previous reports have linked water alkalinity to increased temperature during warm seasons^20^. Our results also indicated a high OD % which might be attributed to the large distance of sample collection from the shore. Overall, our results of water properties were comparable to previous reports of red sea^21^.

Information on the chemical composition of marine organisms is so far restricted to species that have economic impact like mollusks and crustaceans. However, information on gross chemical composition of marine organisms contributes remarkably to our understanding of other species. In the current work, we analyzed the growth biochemical content of *O. dentata*, which were represented as a percentage of the total mass. Our results indicated a higher carbohydrate content, lower protein and nitrogen and a comparable lipid and fiber content compared to other members of Echinodermata^22^. No records of biological analysis of growth biochemical content of *O. dentata* was previously reported. Such difference might be attributed to differences in the environmental factors and specific traits of the studied species.

The identification of purified compounds was done using 1D and 2D-NMR and the chemical structure was confirmed using GC-MS and FT-IR. Our analysis revealed the presence of two purified sesquiterpenoids. The identification of the functional groups of the purified compounds was done using FT-IR by identifying peaks values in the region of IR radiation. Passage of the both compounds through FT-IR, the separation of functional groups is done based on its peaks ratio. The FT-IR results confirmed the presence of O-H and C=C functional groups in both compounds. The only difference between the two compounds is the oxygen bridge between C-11 and C-7 in compound 2. According to analysis results, the two isolated sesquinerpeniods were identified as 8, 11-epoxy-9(15)-himachaladiene-4-ol (O8-ophiocomane) and 7, 11-epoxy-9(15)-himachaladiene-4-ol (O7-ophiocomane). To the best of our knowledge, this is the first time these compounds have been reported according to Science Finder database. Sesquiterpenoids have been associated with significant biological effects including cytotoxic and antimicrobial activities^23,34^.

The search for anticancer agents have been intensified during the last decades. Marine-originated compounds have been investigated and many compounds with cytotoxic, anti-proliferative and anticarcinogenic properties have been isolated from corals^25^, seeweeds^26^, and Ascidiaceans^27^. Our team has successfully extracted two steroids from starfish *Acanthaster planci* with cytotoxic, antibacterial and antidiabetic activities^28^. In the current work, we’ve investigated the cytotoxic activities of *O. dentata* crude extract and purified compounds for cytotoxic activities on both ex vivo and in vivo models. The in vitro cell cytotoxicity assay exhibited that brittle star *O. dentata* purified compounds has a cytotoxic and anti-proliferative activity against breast carcinoma cell line MCF-7 in a dose-dependent manner that is comparable to the chemotherapeutic agent cisplatin. In vivo experiments revealed that the crude extract also exhibited a significant anti-proliferative effect represented in a significant reduction in tumor size in mice when administered either before or after tumor injection. The anti-tumor effect was also evident through histopathological examination. Hematological parameters and liver function and liver histopathological examination also indicated a significant improvement of overall condition of Ehrlich solid tumor-bearing mice treated with Brittle star extract compared to the untreated group.

A number of studies have investigated different Ophiuroids as a source of anti-tumor and anti-metastatic metabolites^29^. Dichloromathane extract of *Ophiocoma* *erinaceus* have been reported to exert a cytotoxic effect that can be used in conjunction with doxorubicin to treat leukaemia cells by inducing apoptosis^30^. A polysaccharide isolated from *Ophiocoma* *scolopendrina* have also shown a potential chemotherapeutic approach for improving paclitaxel effectiveness in the treatment of refractory ovarian cancer^31^. Extracts of *O. erinaceus* and *Ophiomastrix annulosa* have shown cytotoxic activities against MCF -7, VERO and HELA cell lines in a concentration-dependent manner^32^. Purified active components from the North Pacific brittle star *Ophiura sarsii* have showed remarkable cytotoxic activity against triple negative breast cancer cells and might be used as photosensitizers in photodynamic anticancer treatment^33^. The mechanisms by which brittle star extracts exert their anti-cancer effects have been investigated. Recent research has revealed that brittle star *O. erinaceus* extracts have anti-angiogenic and anti-tumor effects by modulating the expression levels of TGF-, VEGF, and bFGF in vascular endothelial cells^34,35^. Brittle star extract have also been reported to exhibit anti-metastatic effects in human cervical cancer cell line (HeLa cells)^36^ as well as anti-proliferative and pro-apoptotic effects on the same cells^34^. *O.* erinaceus extract was reported to trigger intrinsic apoptotic pathway by down-regulation of Bcl2 levels and upregulation of caspase-3 and -9 activities as well as inducing reactive oxygen species generation^37^.

*Ophiocoma dentata* crude extract have shown antimicrobial activity against several bacterial stains including *S. aureus*, *V. damsela* and *P. aeruginosa*, *E. coli*, and *S. faecalis* unlike the pure compounds when exhibited antimicrobial activities against *P. aeruginosa* alone. *Ophiura albida* have also been reported to possess antimicrobial activities as well as α-glucosidase activity that is involved in glucose and starch digestion^38^. Red sea marine organisms have also reported for antimicrobial activity. Soft coral *Paralemnalia thysoides* organic extracts showed activities against. *E. coli*, *C.* albicana and *Aspergillus niger*^39^. Previous studies have attributed the bioactivities of *P. thyrsoides* to new sesquiterpenoids isolated from its ethanol extract^40^.

## Conclusions

The current study has focused on the chemical and bioactivities of Brittle stat *Ophiocoma dentata* collected from the Red Sea. Two newly identified sesquinternoids have been isolated and tested along with the crude extract for cytotoxic and antimicrobial activities. Our results indicated that *O. dentata* represents a promising candidate for identification of new cytotoxic and anti-proliferative activities against MCF-7 cell line and in Ehrlich tumor-bearing mice. These results paves the way for their use as adjuvant anti-cancer agents along with chemotherapeutic drugs without increasing drug side effects. *O. dentata* extract also have shown antimicrobial activities against several human bacterial pathogens which indicate a promising future pharmaceutical application.

## Recommendations

Further investigations on the mechanisms by with *O. dentata* isolated compound exert their cytotoxic and anti-microbial activities are recommended. Exploring the cytotoxic activities of isolated sesquiterpeniods in combination with other therapeutic approaches like chemo and radiotherapy can a promising approach for their pharmaceutical and clinical application.

## Methods

### Study area and water analysis

Brittle star; *O. dentata* were collected using SCUBA diving at the month of June, at 1-10 m depth range during low tide from Hurghada (Latitude: 27.28°N and Longitude: 33.77°E). Water Samples from the study area was collected and hydro-chemical and physical properties were measured including temperature (standard Schmidt thermometer), salinity (Beckman induction Salinometer), pH (Orion pH meter), DO (Winkler technique), OOM (FAO technique), Ca levels and alkalinity^41,42^.

### Sample collection and content biochemical analysis

The specimen were identified according to their morphological characteristics in the National Institute of Oceanography and Fisheries using the Manual of Taxonomy of Echinodermata as a reference^43^. Each sample was washed with water to remove dirt and sand. To remove dirt and sand, each sample was washed in water. Samples were frozen on-site in polypropylene bags before being transported to the laboratory for extraction. The fresh animals were chopped finely into small pieces until a homogeneous gelatinous mass is formed. For biochemical analysis, samples were dried at room temperature in darkness and stored in packages. Humidity was measured by calculating the reduction in mass after drying a 5 mg of sample at 100 °C, ash content was obtained by measuring the residual in sample after heating for 600 °C. Organic matter was determined by calculating the difference between 100% and sum of humidity and ash values^44^. Nitrogen and protein were determined by sample digestion, neutralization followed by titration^45^. Lipid content was determined by extraction with ethyl ether in Soxhlet for 5 h. the solvent was then evaporated and the residual mass was measured as lipid content. For fiber content determination 5 g of sample were digested with 5% HCl for, filtered, washed, followed by digestion with 5% NaOH under reflux, filtered and washed until negative alkaline reaction was obtained. The product was then dried and weighed^44^. Carbohydrate content was calculated at a percentage by subtraction of humidity, protein, lipids, fibers and ash.

### Sample extraction and isolation

The samples were extracted with chloroform and methanol at room temperature. The sample weight/solvent volume ratio used was 1/2. Extraction was done (3 times) for one week, the crude extract was filtered by Whatman filter paper no.1 (Sigma-Aldrich, St. Louis, CO, USA) and the filtrate was concentrated by rotary evaporator (Stuart RE300, Bibby Scientific Ltd.) at 40 °C under reduced pressure. The resultant residue was combined to obtain 6.25 mg of crude extract which was kept frozen at −20 °C for analyses of biological activities.

### Extract purification and isolation of fractions

Purification of the crude extracts was done using column chromatography technique. The crude extract was chromatographed on silica gel size 60–200 µm (70–230 mesh) as a stationary phase and elution with increasing polarity of the mobile phase (i.e. starting from 100% Hexane through Hexane-DCM mixture, DCM-methanol mixture till 100% methanol) under atmospheric pressure. Two of the obtained fractions (1 and 2) were further purified by a secondary chromatography column with silica gel size of 40–30 µm (230–400 mesh). Preparative thin layer chromatography (TLC) was then performed on fraction 2 using aluminum plates impregnated with silica gel 60 F_254_ (20 × 20 cm). The developed spots were detected under ultraviolet light detector lamp at 254 nm for detection of ultraviolet active compounds which contain unsaturation system (double bonds or benzene ring), followed by spraying with sulfuric acid and heating on a hot plate at 120 °C for detection of other pure compounds.

### Isolated secondary metabolites’ structure elucidation (pure compounds)

All of the pure chemicals were subjected to spectroscopic techniques such as mass spectrometry (MS), nuclear magnetic resonance (NMR), and infrared (IR) spectroscopy.

#### NMR analysis

The measurement of (^1^H) NMR spectra were recorded at Bruker AVANCE 400 MHZ NMR spectrometers. And measurement of (^13^C) NMR spectra was recorded at 100 MHZ. COSY, HSQC, HMBC, and NOESY were also done. Deuterated chloroform solvent (CDCl_3_) was used to dissolve samples for NMR measurement. The selection of the solvent was dependent on the solubility of the sample. The observed chemical shift (δ) values were given in *ppm* and the coupling constants (J) in *Hz*.

#### FT-IR analysis

Infrared spectroscopy was used to identify the functional groups and validate the structure of the pure molecule. Dried pure chemicals were ground to a fine powder and combined 1:1 with dried KBr powder to form compressed pellets, which were then analysed using a Bruker Alpha FTIR spectrometer. On a Bruker Alpha Fourier transform infrared spectrometer, spectra were acquired in the absorbance mode from 4000 to 400 cm^−1^.

#### GC–MS analysis

The pure compounds were subjected to GC–MS analysis to identify and confirm the structures of various purified bioactive compounds. The sample was analyzed in an Agilent 7890A 5975C gas chromatography Mass spectroscopy instrument. The carrier gas was Helium and the analysis was performed at 90–300 °C. The identification and confirmation of the structure of pure compounds was done using computer matching of mass spectra with those of standards.

### Cytotoxicity and anticancer activity

#### In vitro cytotoxicity study

*Cell culture:* Human breast adenocarcinoma MCF-7 cell line (ATCC HTB-22™) was maintained in DMEM/F12 (Lonza Group, Ltd.) supplemented with 10% fetal bovine serum (Sigma-Aldrich, Merck KGaA), 100 IU/ml penicillin and 100 mg/ml streptomycin. Cells were cultures in a humidified atmosphere at 37 °C with 5% CO_2_. Cells were examined under an inverted microscope after 48 h. MCF-7 cells formed a confluent adherent layer (80% confluence). After completion of the treatment protocol, MCF-7 cells were harvested using 0.05% trypsin and 0.02% EDTA.

*Treatments of MCF-7 cells*: MCF-7 cells were treated with Compound 1, Compound 2 or Cisplatin. Stock solutions of the test compounds were dissolved in DMSO, diluted with culture medium and stored at −20 °C. Serial dilutions were made in culture medium and cells were treated with final concentrations of 3.75, 7.5, 15, 30, 60 and 120 µM. Control group was treated with media with 0.1% DMSO. Following treatment,cells were incubated for 48 h at 37 °C in 5% CO_2_ humidified incubator. All experiments were carried out in triplicates and repeated three times. The color intensity is correlated with the number of healthy living cells.

*MTT Cell viability assay:* The viability of MCF-7 cells was evaluated using 3-(4, 5-dimethylthiazol-2-yl)-2, 5-diphenyl Tetrazolium bromide (MTT) assay^46^. After treatment, cells were incubated with 100 μl of MTT (5 mg/10 ml basal medium DMEM) was added to each well and incubation continued at 37 °C for 2 h. The medium was then carefully removed, 150 μl DMSO was added to each well and the absorbance of solubilized blue formazan read at wavelength of 490 nm using a microplate reader and cell survival was calculated using the formula: Viability (%) = (Absorbance of sample/Absorbance of control) * 100. The LC_50_ values were determined using linear regression.

#### In vivo study using laboratory animal

The brittle star; *O. dentata* crude extract was tested for anticancer activity in Ehrlich carcinoma bearing mice. The experiment was performed according to ethical guidelines on animal experimentation and was approved by Institutional Animal Care and Use Committee of the University of Alexandria (#0122081231).

*Preparation of Ehrlich Solid Tumors:* Ehrlich ascites carcinoma cells were sustained by intraperitoneal transplantation in host animals every 10 days. The ascetic fluid was harvested on the 3rd to 5th day after inoculation, washed with PBS, pH 7.4, centrifuged at 200 g, washed again then assessed for cell viability using trypan blue stain^47^. For the transplantation of solid tumors, cells were re-suspended in saline to a final concentration of 1 × 10^6^/100 μl. Cells were then injected subcutaneously on the hind left thigh according to the experimental design below and observed for the development of solid tumors. The tumor size was measured using a digital caliper (1–150 mm) every other day and the tumor volume was calculated using the formula (V= ½ × (D × d^2^), were; V is the tumor volume, D is the higher diameter and d is the lower diameter^48^.

*Animal Groups and Treatment:* A total of 60 Swiss albino mice (age 8–10 weeks, mean weight 20 ± 2 g) were used in this experiment. Mice were housed under standardized conditions (22 ± 2 °C, 12h dark/12 h light cycle, 55–65% humidity and access to food and water). The mice were randomly divided into 4 equal groups as follows: negative control group, which included mice kept in normal healthy condition, positive control group, included Ehrlich solid tumor-bearing mice, curative group, which included Ehrlich solid tumor-bearing mice treated with intraperitoneal injection of 100 µl of the crude extract (50 µg/ml) twice a week for about 3 weeks, and preventive group, which included normal mice treated with intraperitoneal injection of 100 µl of the crude extract (50 µg/ml) twice a week for about 3 weeks prior to the development of Ehrlich solid tumors. The time of injection, tumor mass size and behavioral changes and fatalities were recorded daily.

*Sampling, biochemical and hematological Investigations:* Blood samples were withdrawn from carotid artery of mice into EDTA-containing tubes under mild anesthesia. Each sample was divided into 2 parts, the first was used for red blood cells, white blood cells and platelets count using Beckman Coulter DxH hematology analyzer. The second part of each sample was centrifuged at 4000 g for 10 min and the plasma was aspirated into Eppendorf tubes and stored at −80 °C until used for the assessment of liver functions (SGOT and SGPT) (Beacon Diagnostics PVT, Ltd.).

*Histopathological Examination:* At the end of the experiment, mice were sacrificed under diethyl ether anesthesia. Fresh tumor specimens were collected and fixed using 99% ethyl alcohol for 24 h then embedded in paraffin using with Vacuum Rotary VRX-23 (Sakura Finetek Japan Co., Ltd., Tokyo, Japan), sectioned and stained with hematoxylin and eosin for histopathological examination.

### Antimicrobial activity

Antibacterial activity was determined against cultures of: *S. aureus* (Gram +ve, ATCC 25923)*, E. coli* (Gram –ve, ATCC 25922)*, V. damsela* (Gram –ve, ATCC 33539)*, P. aeruginosa* (Gram –ve, ATCC 27853)*, A. hydrophila* (Gram –ve, ATCC 35654), and *S. faecalis* (Gram +ve, ATCC 29212), whereas antifungal activity was determined against *C. albicans* (ATCC 10231).

The Dauer Kirby test^49^ was used to perform the agar diffusion assay. For the crude extract and pure components, aliquots of the test solution were applied to sterile filter paper discs (6 mm diameter) with a final disc loading concentration of 200 mg/ml. The impregnated discs, together with discs containing solvent blank and positive control ciprofloxacin, were put on Tryptic soy agar plates that had already been seeded with the designated test organisms. Antimicrobial activity was measured as distinct zones of inhibition surrounding the discs after 24 h of incubation at 37 °C. The experiment was repeated three times, with the average values reported. The antimicrobial activity was expressed as absolute activity unit (AU) which equals π Y2/π X2 where: Y refers to Inhibition Zone diameter and X refers to Disc diameter. A distinct inhibitory zone with a diameter of at least 1 mm was considered positive.

## Supplementary Information

Supplementary Information.
