# Supplementary material for: Cytotoxic and antimicrobial activities of two new sesquiterpenoids from red sea brittle star Ophiocoma dentata
Source: Sci Rep. 2022 May 17;12:8209. doi: 10.1038/s41598-022-12192-7 (PMC9114132; doi:10.1038/s41598-022-12192-7)

Cytotoxic and Antimicrobial Activities of Two New Sesquinterpenoids from Red Sea Brittle Star Ophiocoma dentata

Shaymaa E. El Feky [1,*], Mohamed S.M. Abd El Hafez [2], Nadia A. Abd El Moneim [3], Hassan A.H. Ibrahim [2], Mohamed A. Okbah [2], Athar Ata [4], Amel S. El Sedfy [5], and Ahmed Hussein[6]

[1]Radiation Sciences Department, Medical Research Institute, University of Alexandria, Egypt.
[2] National Institute of Oceanography and Fisheries, NIOF, Cairo, Egypt.
[3]Cancer Management and Research Department, Medical Research Institute, Alexandria University, Egypt.
[4]Department of Chemistry, University of Winnipeg, Winnipeg, MB, Canada.
[5]Patholohy Department, Medical Research Institute, University of Alexandria, Egypt.
[6]Department of Biotechnology, Institute of Graduate Studies and Research, Alexandria University, Egypt.
[*]shaymaa.elfeky@alexu.edu.eg

Contents of Supporting Information

No.	Contents	P
1	Table S1.^{1}H and ^{13}C NMR data of compound **1&2** (δ in ppm, J in Hz)	2
2	Figure S1. ^{1}H NMR spectrum of compound (**1**)	3
3	Figure S2. ^{13}C NMR spectrum of compound (**1**)	4
4	Figure S3. HSQC spectrum of compound (**1**)	5
5	Figure S4. HMBC spectrum of compound (**1**)	6
6	Figure S5. COSY spectrum of compound (**1**)	7
7	Figure S6. NOESY spectrum of compound (**1**)	8
8	Figure S7. ^{1}H NMR spectrum of compound (**2**)	9
9	Figure S8. ^{13}C NMR spectrum of compound (**2**)	10
10	Figure S9. HSQC spectrum of compound (**2**)	11
11	Figure S10. HMBC spectrum of compound (**2**)	12
12	Figure S11. COSY spectrum of compound (**2**)	13
13	Figure S12. NOESY spectrum of compound (**2**)	14
14	Figure S13. Brittle star; O. dentate under investigation	15

Table S1. ^{1}H and ^{13}C NMR data of compounds **1&2** (δ in ppm, J in Hz)

	1		2	
**position**	δ_H (J in HZ)	δ_C mult.	δ_H (J in HZ)	δ_C mult.
**1**	1.88 (d,4.0)	50.3	1.44 (d,4.0)	50.7
**2**		35.7		35.4
**3**	2.07 (d,5.2)	43.9	1.77 (dd, 12.0, 4.0)	43.9
**4**	4.47 (dd,4.0,4.4)	67.8	4.31 (m)	67.8
**5**		40.3		38.5
**6**	2.11 (m) overlap	22.6	2.15 (m) overlap	22.6
**7**	4.74 (dd,4.4,5.2)	68.3	2.15 (m) overlap	23.6
**8**	2.17 (m) overlap	25.4	4.57 (dd, 4.0, 8.0)	70.4
**9**		145.7		146.6
**10**	2.02 (s)	37.1	2.21 (s)	36.0
**11**		63.4		68.5
**12**	0.98 (s)	33.5	0.98 (s)	33.7
**13**	1.17 (s)	29.6	1.19 (s)	29.6
**14**	1.56 (s)	23.5	1.28 (s)	22.6
**15**	4.89 (s)<br>5.28 (s)	114.8	4.85 (s)<br>5.18 (s)	113.6

Figure 1. ^{1}H NMR spectrum of compound (**1**)

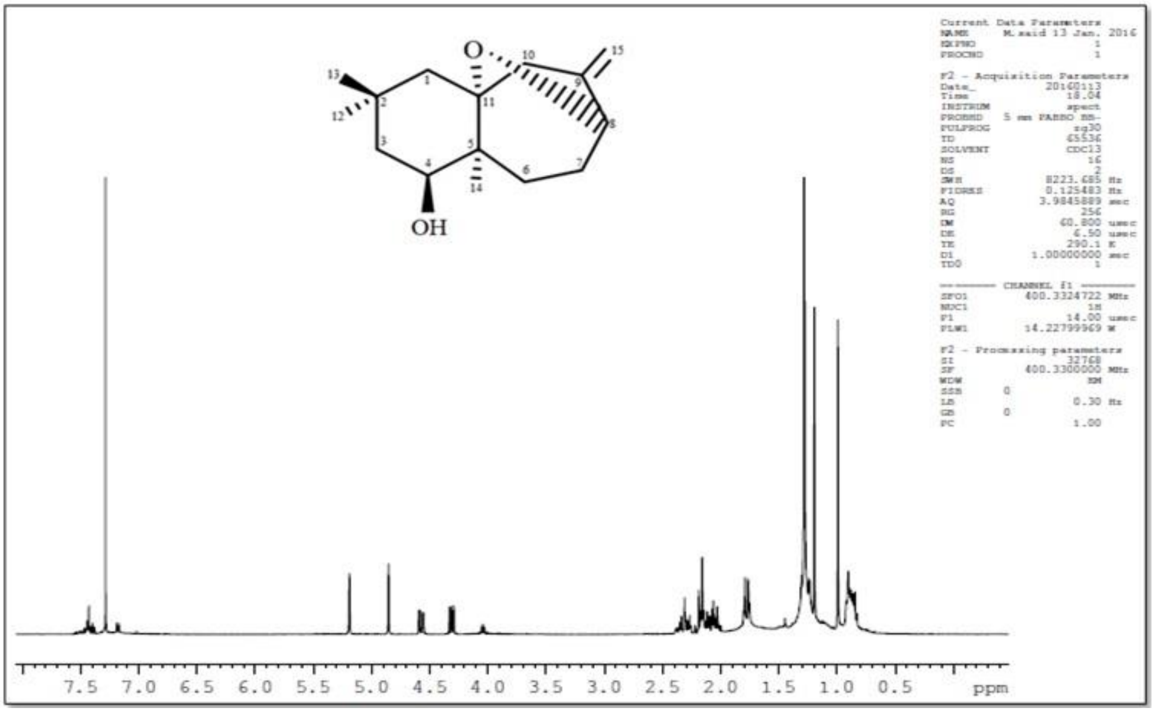

Figure 2. ^{13}C NMR spectrum of compound (**1**)

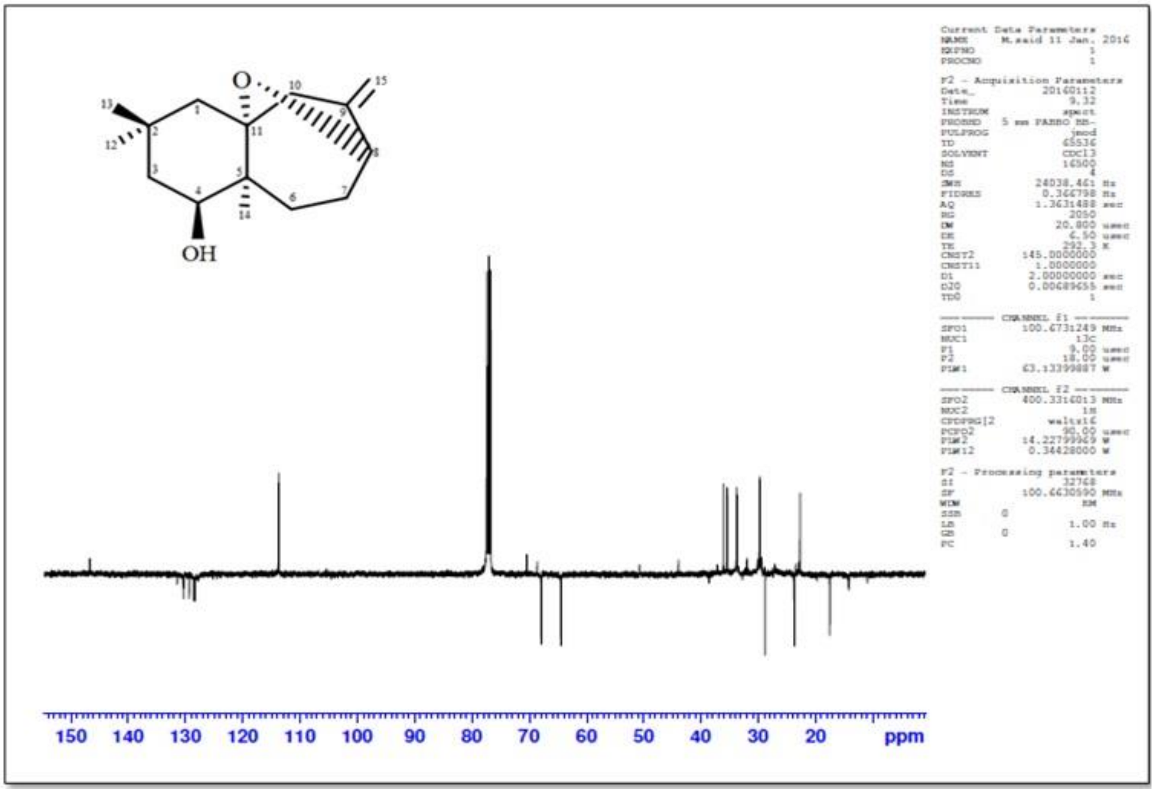

Figure 3. HSQC spectrum of compound (**1**)

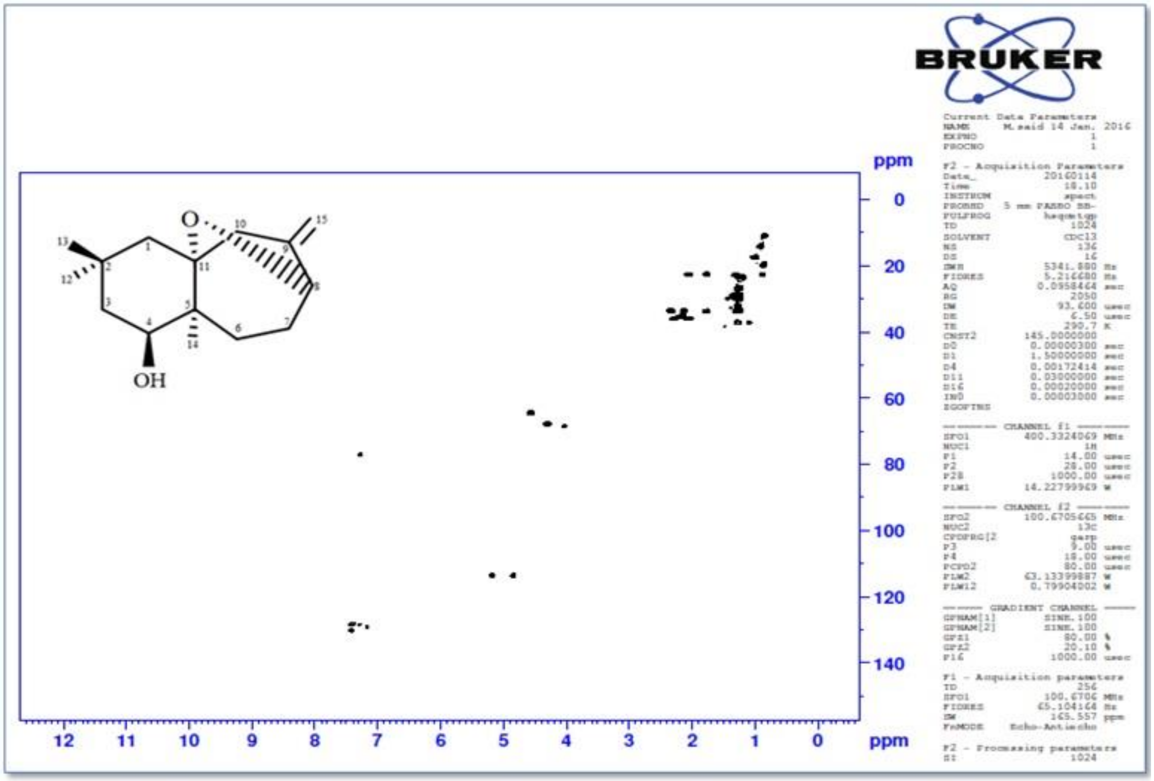

Figure 4. HMBC spectrum of compound (**1**)

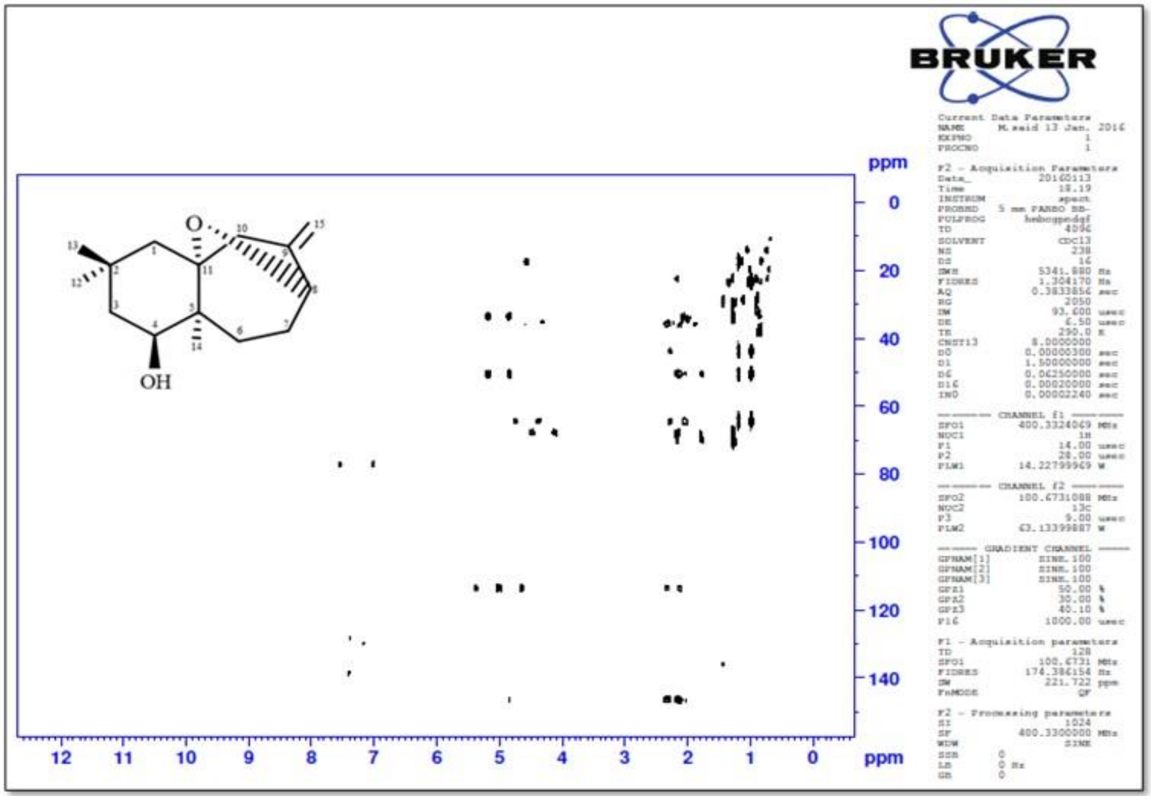

Figure 5. COSY spectrum of compound (**1**)

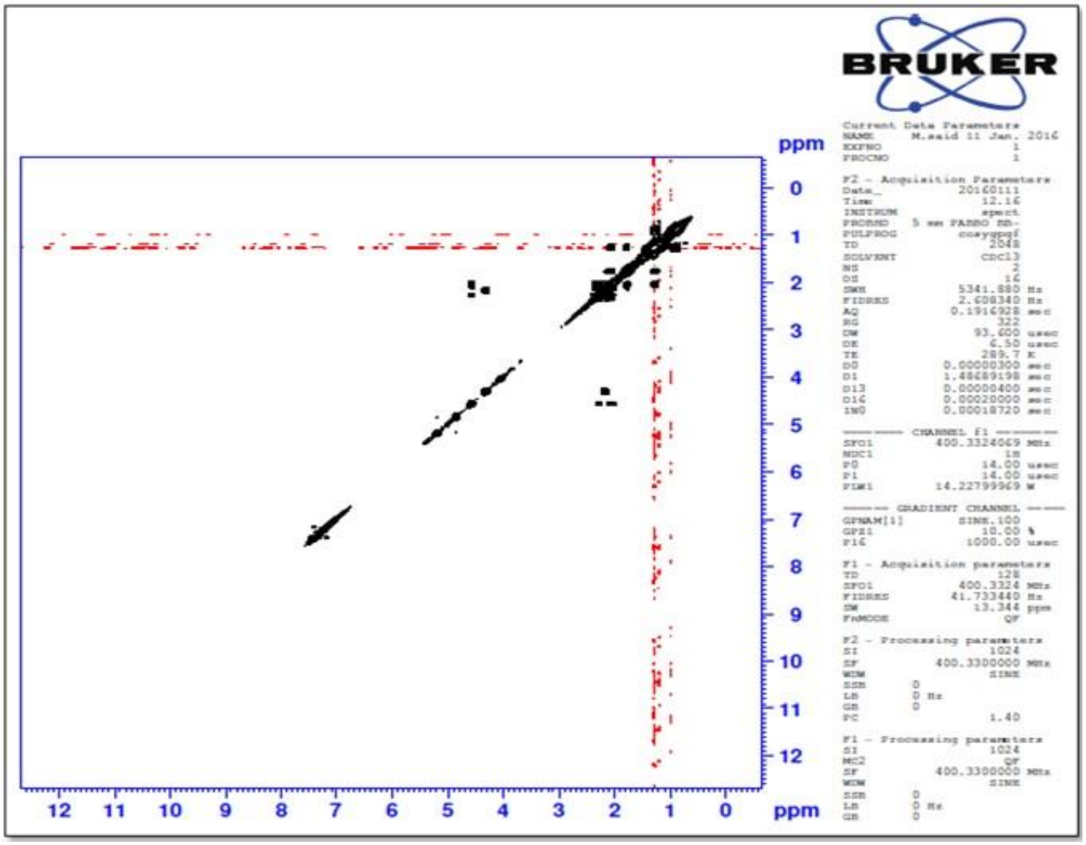

Figure 6. NOESY spectrum of compound (**1**)

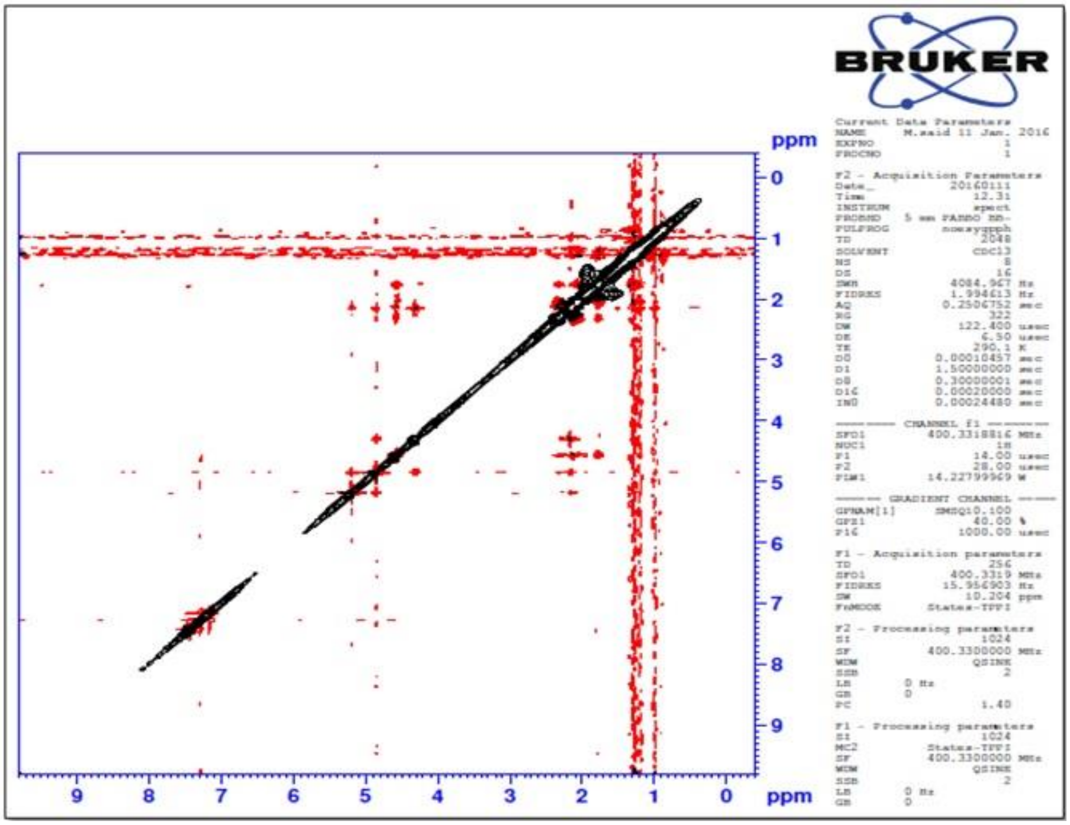

Figure S7. ^{1}H NMR spectrum of compound (**2**)

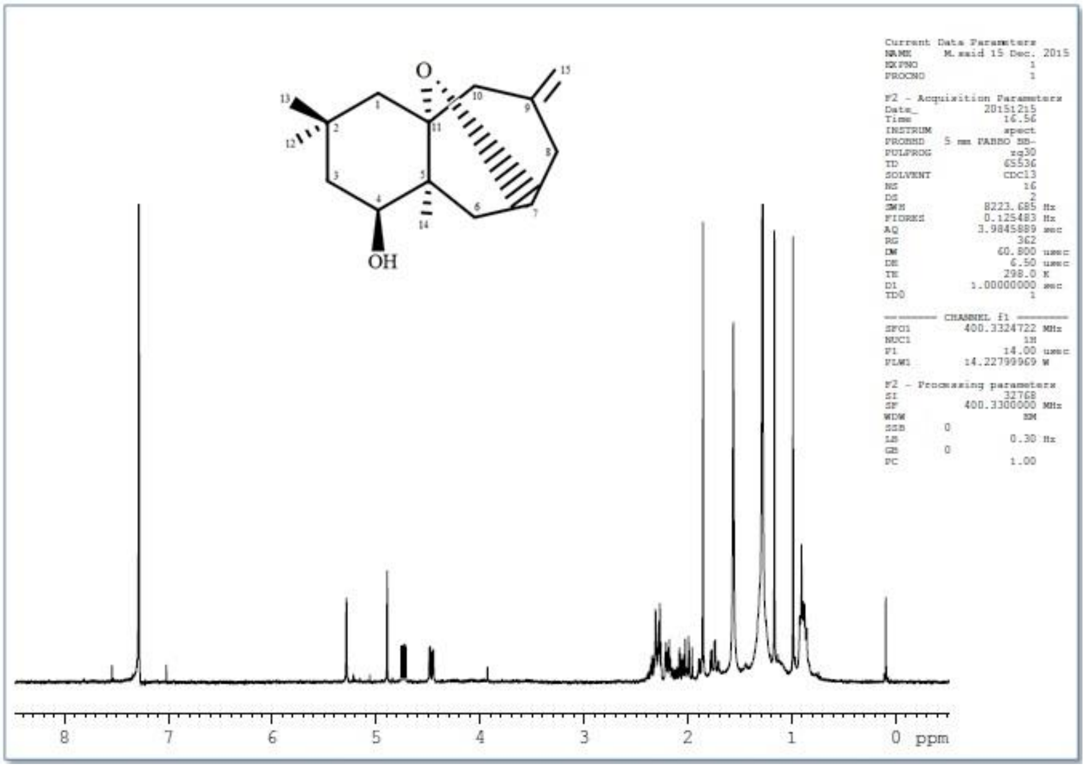

Figure S8. ^{13}C NMR spectrum of compound (**2**)

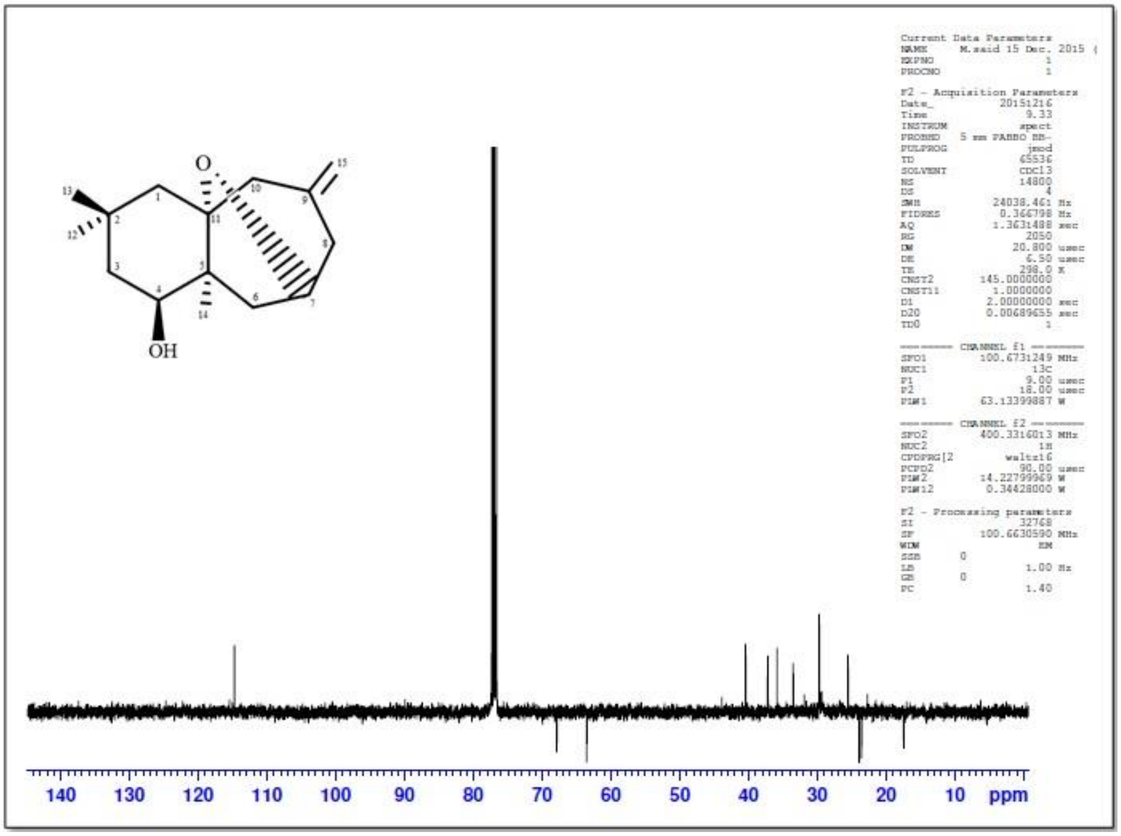

Figure S9. HSQC spectrum of compound (**2**)

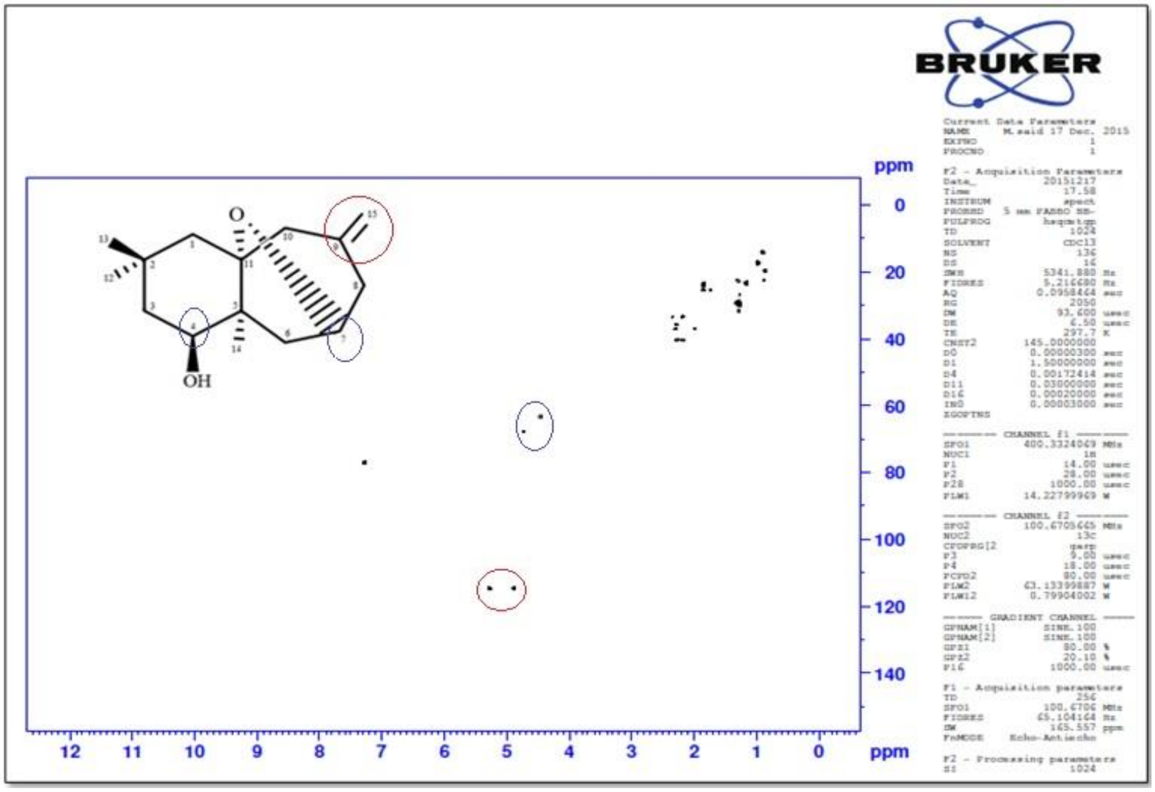

Figure S10. HMBC spectrum of compound (**2**)

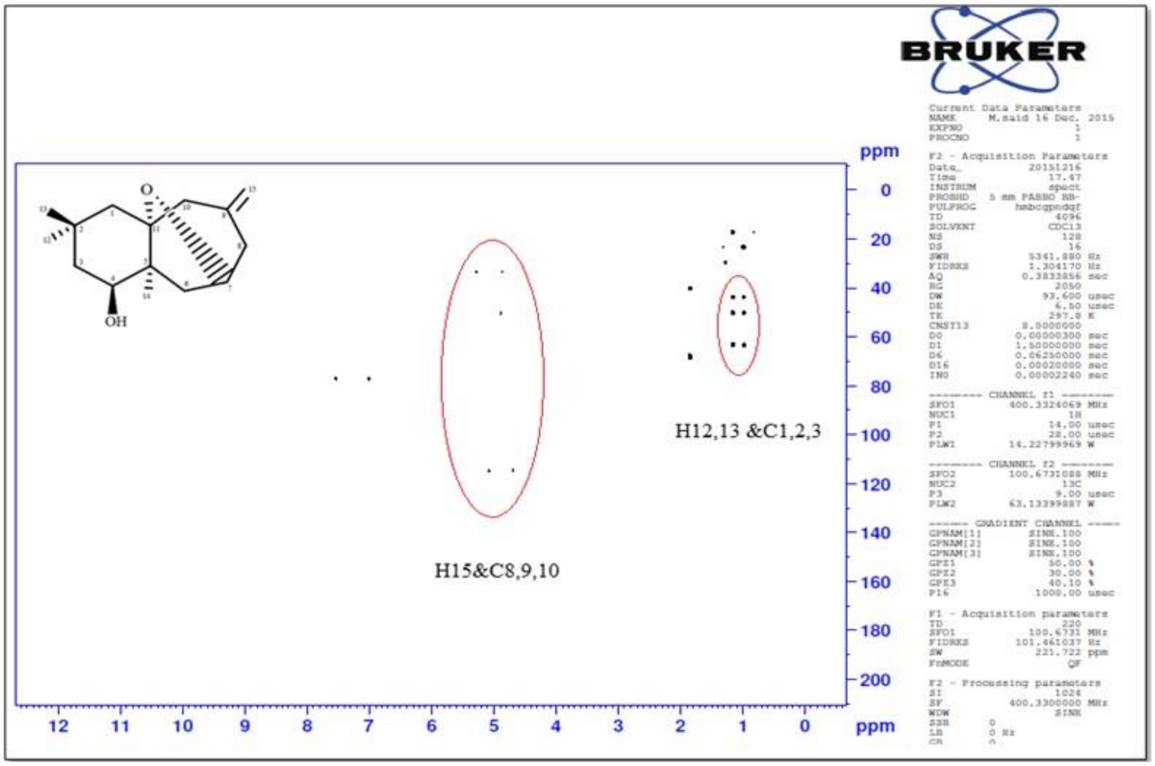

Figure S11. COSY spectrum of compound (**2**)

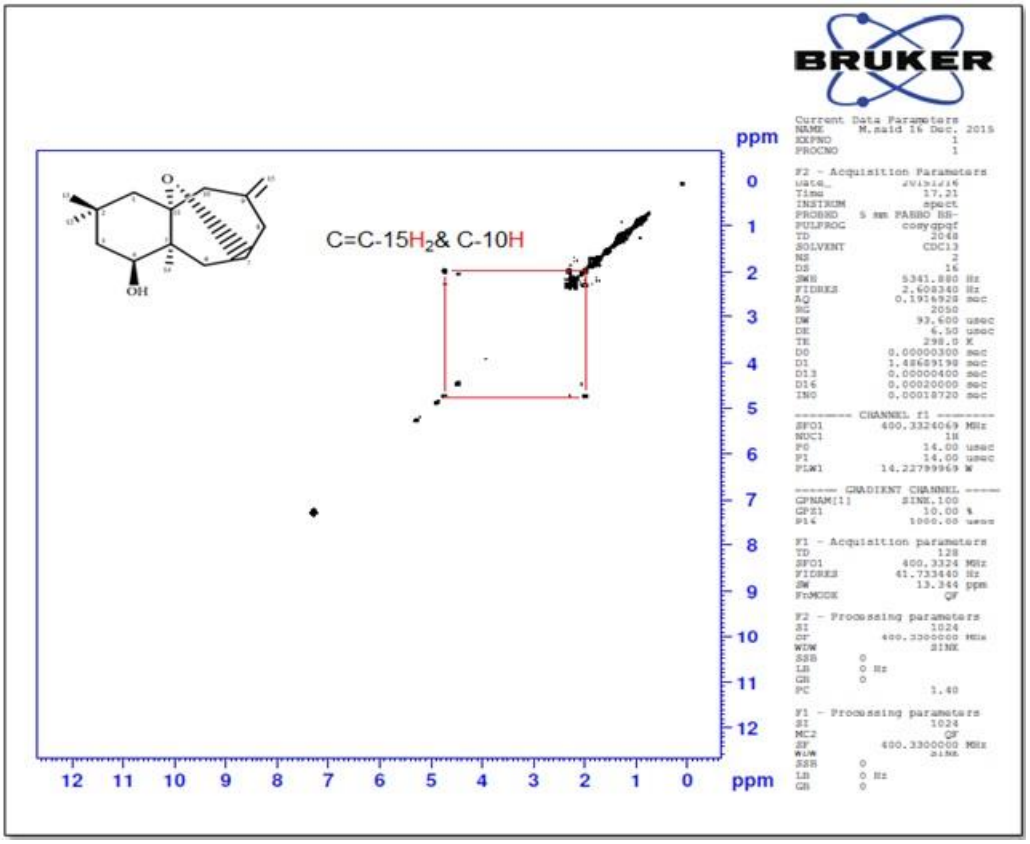

Figure S12. NOESY spectrum of compound (**2**)

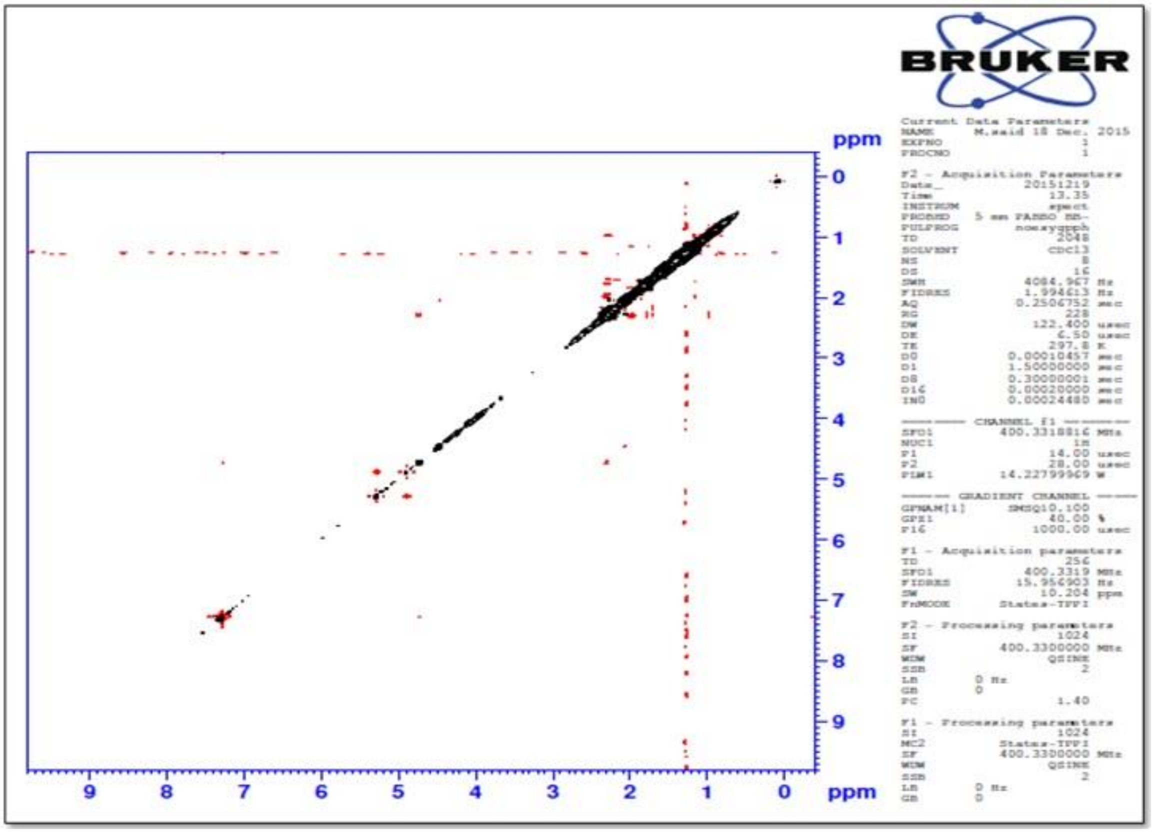

Figure S13. Brittle star; O. dentate under the current investigation

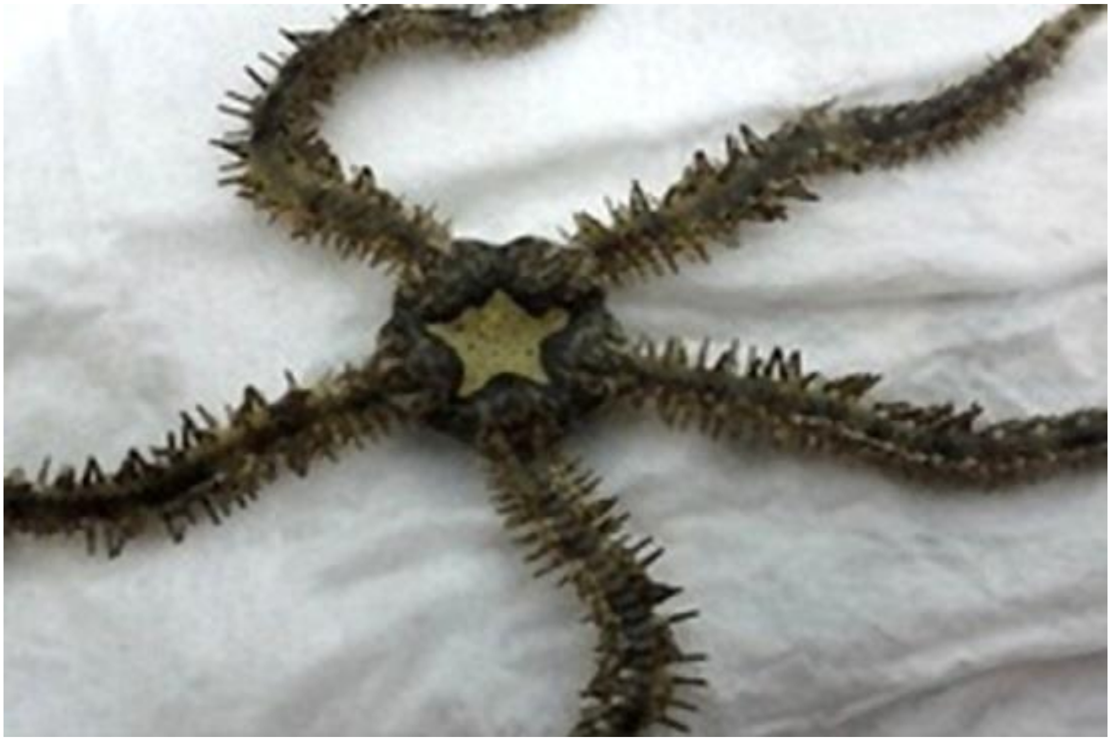

Supplement: Supplementary file 1 — Supplementary Information. [file 41598_2022_12192_MOESM1_ESM.pdf]
